# Strategies for analyzing highly enriched IP-chip datasets

**DOI:** 10.1186/1471-2105-10-305

**Published:** 2009-09-22

**Authors:** Simon RV Knott, Christopher J Viggiani, Oscar M Aparicio, Simon Tavaré

**Affiliations:** 1 Molecular and Computational Biology Program, University of Southern California, Ray Irani Hall, University Park Campus, Los Angeles, CA, 90089-2910, USA

## Abstract

**Background:**

Chromatin immunoprecipitation on tiling arrays (ChIP-chip) has been employed to examine features such as protein binding and histone modifications on a genome-wide scale in a variety of cell types. Array data from the latter studies typically have a high proportion of enriched probes whose signals vary considerably (due to heterogeneity in the cell population), and this makes their normalization and downstream analysis difficult.

**Results:**

Here we present strategies for analyzing such experiments, focusing our discussion on the analysis of Bromodeoxyruridine (BrdU) immunoprecipitation on tiling array (BrdU-IP-chip) datasets. BrdU-IP-chip experiments map large, recently replicated genomic regions and have similar characteristics to histone modification/location data. To prepare such data for downstream analysis we employ a dynamic programming algorithm that identifies a set of putative unenriched probes, which we use for both within-array and between-array normalization. We also introduce a second dynamic programming algorithm that incorporates *a priori *knowledge to identify and quantify positive signals in these datasets.

**Conclusion:**

Highly enriched IP-chip datasets are often difficult to analyze with traditional array normalization and analysis strategies. Here we present and test a set of analytical tools for their normalization and quantification that allows for accurate identification and analysis of enriched regions.

## Background

Chromatin immunoprecipitation on tiling array (ChIP-chip) studies attempt to identify genomic features such as protein binding [1,2] or histone modification/occupancy [3,4]. In the former, the regions of interest are generally small, resulting in a low proportion of enriched probes and the data can be considered to come from one of two distributions, enriched or non-enriched. In contrast, the regions analyzed in the latter studies are generally large and can have multiple levels of enrichment within and between them, making their analysis more difficult. Bromodeoxyuridine immunoprecipitation on tiling array (BrdU-IP-chip) datasets, which map recently replicated regions of the genome, have characteristics that are similar to histone modification/occupancy experiments. While computational tools have been developed to address the analytical issues associated with mRNA-chip and protein binding ChIP-chip studies, the highly enriched IP-chip datasets described above pose unique problems requiring new investigative strategies. In a recent study we used BrdU-IP-chip to investigate the effects of chromatin modifications on replication timing/efficiency in *S. cerevisiae *cells [5]. We have developed a new set of computational tools for the normalization and analysis of these and similar experiments and we present them here.

5-Bromo-2'-deoxyuridine (BrdU) is a synthetic thymidine analog that pairs with deoxyadenosine and, when available to the cell, is incorporated into replicating DNA at positions normally occupied by deoxythymidine. After genomic DNA is extracted from a cell culture, regions that have been replicated in the presence of the molecule can be extracted by centrifugation or with BrdU-specific antibodies. In [6,7] BrdU-incorporated DNA was separated by isopycnic centrifugation and run on Affymetrix tiling arrays to analyze human cell replication profiles. In [8] BrdU-IP DNA samples from both early and late S-phase were fluorescently labeled and co-hybridized on two-color arrays to analyze the replication timing dynamics of the *Drosophila *genome. Here we concentrate specifically on the BrdU-IP-chip assay, which involves the labeling and co-hybridization of BrdU-IP and genomic DNA on two-color tiling arrays. In [9,10] this procedure was employed to study the co-localization of replication forks with various DNA binding factors. In [11] the authors used BrdU-IP-chip to investigate differences in replication fork progression in response to intra-S checkpoint activation in *S. cerevisiae*. More recently, this technique has been employed in a comparative genome-wide analysis of replication activity throughout various stages of embryonic stem cell differentiation [12].

Analyses of BrdU-IP-chip experiments aim to distinguish true biological signals (DNA replication activity) from array noise and to examine those signals for magnitude and associated genomic features. Microarray datasets (specifically from two-color platforms) typically contain errors resulting from sample handling, preferential amplification and labeling bias, making this task difficult. In attempts to correct for this, several ChIP-chip studies have incorporated mock controls into their experimental design [3,13]. Under this protocol, for each experiment a mock sample (DNA acquired with a non-specific antibody or no antibody at all) is hybridized against the same total DNA as the experimental sample. Following array quantification, true positive signals are identified as those that are significantly higher in the experimental data than the mock data. Recently, it has been shown that without these controls the false positive rate can be high [3]. Unfortunately, the use of these controls significantly increases the cost of each experiment and furthermore, the strategy fails to address issues pertinent to studies aimed at comparing the magnitude of signals across different experimental conditions.

Computational alternatives to the use of mock controls have been developed to work with two-color array data. These typically involve a within-array normalization step aimed at eliminating intensity bias (where *M *= log_2_(IP/Total) values show dependence on their corresponding *A *= (log_2_(IP) + log_2_(Total))/2 values) and can be followed by a between-array normalization step to remove location and scale variation across multiple experiments [14-17]. Simple loess normalization is usually used in mRNA-chip studies for within-array normalization, based on the assumption that the M-values should follow a symmetric distribution [14,15,17]. Briefly, probes are plotted in the MA plane and a loess curve is fitted to the data. To remove the intensity bias, the resultant curve is then subtracted from the probe M-values.

While mRNA-chip M-values typically follow a symmetric distribution, array studies involving chromatin immunoprecipitation are often associated with asymmetric empirical M-distributions [18]. To remove the intensity bias in ChIP-chip data Peng *et al*. [18] proposed a two-step process in which an initial data transformation is performed under the assumption that chromosomally neighboring probes should have minimal difference in their M-values (with the exception of probes bordering bound and unbound regions). Probes are first plotted in the *δ *(*M*) vs. *δ *(*A*) plane, where *δ *(*M*) and *δ *(*A*) values are the differences between the M- and A-values of neighboring probes, respectively. Under their assumption, when plotted in this plane probe data should have a slope equal to zero. With this in mind, the line of best fit to the probes in this plane is taken as the x-axis for a modified MA plane into which the probes are transformed; we refer to this line as the rotation line. Following this, a modified loess normalization step is performed where the loess curve is fitted to data points within two standard deviations of the median.

If comparisons are to be made across experiments after within-array normalization, between-array normalization is typically applied to remove differences between the empirical M-distributions of the arrays not attributable to true biological variation. For ChIP-chip data, Yang et al. [14] proposed scale normalizing by a value proportional to the median absolute deviation (MAD). Others have proposed quantile normalization [15,16], which forces the M-values of all experiments to follow the same empirical distribution.

Here we demonstrate that current methods for normalizing ChIP-chip datasets may be unsuitable for BrdU-IP-chip experiments, and we describe a novel algorithm for within-array normalization that is robust to the nuances of protein binding and histone modification/occupancy ChIP-chip and BrdU-IP-chip datasets. For each experiment, the algorithm identifies a subset of putative background probes and uses it to transform the data onto a plane where the intensity bias of the dataset is low. We then employ these subsets in between-array normalization and peak identification strategies to prepare the data for downstream analysis. Finally, we present a dynamic programming algorithm that first identifies the optimal alignment of enriched regions across experiments and then assigns these regions to the known and/or predicted origins from which they most likely emanate. This results in more accurate comparisons across experiments and also allows a precise analysis of the chromosomal features surrounding each interesting region.

We illustrate the strategies proposed here on four replicate wild-type (WT) and four replicate mutant *S. cerevisiae *BrdU-IP-chip datasets described in greater detail in [5]. The mutants are *rpd*3Δ cells (Rpd3 is a histone deacetylase) that were shown to have earlier replication initiation (replication fork formation) at a subset of replication origins [19]. All datasets were produced when DNA was harvested from cells one hour after release from *α*-factor (a mating pheromone that arrests cells at the G1-S transition) into hydroxyurea (HU, a chemical that depletes deoxynucleotides and thereby inhibits replication early in S-phase) and BrdU. The well-studied replication landscape of WT *S. cerevisiae *cells in HU and the subset of origins whose altered replication activity in *rpd*3Δ cells is known allows us to test the signal identification and quantification capabilities of our methods in the context of cross-experiment analysis.

## Results and Discussion

### Within-Array Normalization

**Figure 1:**
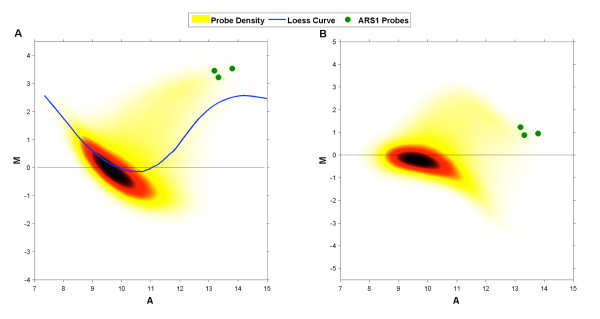
**Testing Loess Normalization**. Illustration of loess normalization for BrdU-IP-chip data. **(A) **The density of all WT probes on the MA plane (red) before normalization (probes within ARS1 are denoted with green dots). During loess normalization a loess curve is fitted to the probes in this plane. **(B) **Probes on the MA plane after the loess curve has been subtracted from their M-values. Note that M-values of ARS1 probes have been pulled towards 0.

To remove the intensity bias present in the BrdU-IP-chip data (Figure 1A) we first attempted simple loess normalization with default parameter settings. Figures 1A and 1B show the result of this normalization on the "cleanest" (as measured by autocorrelation of probe M-values along the genome; cf. [20]) WT dataset. Under the assumption that in the presence of HU earlier and more-efficient origins fire in a higher percentage of cells than do later less-efficient origins, we expect that the amount of IP DNA, and thus M-values, associated with active origins will have larger magnitudes than those associated with less active origins. The green points on the MA plots signify probes within ARS1 (an origin that fires early and efficiently in HU [21]) and these can be used as a measure of the normalization procedure's performance. Due to the high percentage of BrdU-enriched probes the loess curve is pulled away from the background probe set (non-BrdU-enriched probes) during fitting. As a result, when these curves are used for normalization they artificially lower the M-values of some significantly BrdU-enriched probes (e.g. probes within ARS1).

**Figure 2:**
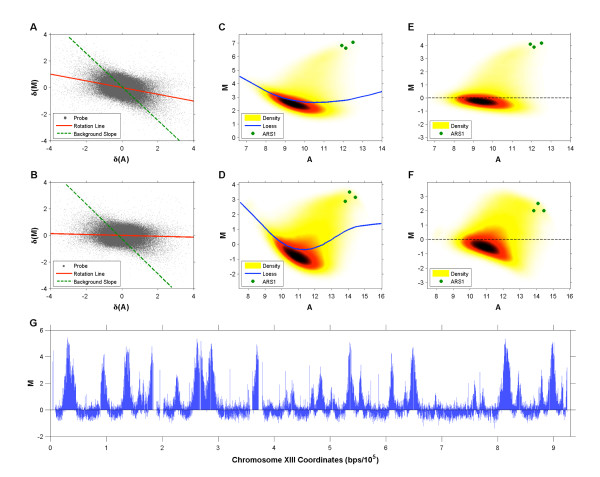
**Testing ChIP-chip Normalization Methods**. Illustration of method proposed in [18] for normalization of BrdU-IP-chip data. Each probe in the WT **(A) **and *rpd*3Δ **(B) **datasets is plotted in the *δ*(*M*) vs. *δ*(*A*) plane and a line of best fit, which should run parallel to the slope of the background distribution, is identified. The WT **(C) **and *rpd*3Δ **(D) **probes transformed onto the modified MA plane with probes from within ARS1 highlighted (green). Following this transformation a loess curve is fitted to probes within 2 standard deviations of the median M-value. WT **(E) **and *rpd*3Δ **(F) **probes after the final loess normalization step. **(G) **Raw M-values of WT probes plotted in the chromosomal plane (chromosome XIII shown here).

Next we applied the two-step within-array normalization scheme for ChIP-chip data proposed in [18] to BrdU-IP-chip data, again using default parameter settings. Figures 2A and 2B show the probes of the "cleanest" WT and *rpd3Δ *datasets, respectively, plotted in the *δ *(*M*) vs. *δ *(*A*) plane. The rotation lines identified in this plane do not follow the slope of the background distribution in the MA plane. After probes have been transformed using these lines, a residual intensity bias remains that seems to be more prominent in the *rpd*3Δ data (Figures 2C &2D). Unfortunately this residual bias appears significant enough to affect the modified loess step, resulting in a normalized probe set with characteristics similar to probes after simple loess normalization (a sloping background distribution and artificially lowered ARS1 probe M-values, Figures 2E &2F). When these methods are applied to a slightly "noisier" (as measured by autocorrelation once more) *rpd*3Δ dataset, they define a rotation line whose slope has the opposite sign to that of the background distribution (see Additional file 1), leading to a more obviously incorrect transformation.

The methods proposed in [18] were developed under the assumption that probe M-values follow one of two distributions (enriched or non-enriched) and that these distributions have relatively low variance (i.e., enriched probes have similar M-values). While this assumption is generally valid for ChIP-chip data, it does not hold for BrdU-IP-chip experiments. Figure 2G shows that the replicated regions are wide (up to 30 kbp) and, due to the asynchrony of replication fork movement across the cell population, there is no sharp boundary between enriched and non-enriched regions, but rather an incremental decrease in M-values on either side of each peak apex. We suggest that these characteristics, in not following those of typical ChIP-chip data, are the reason why the method proposed in [18] is sub-optimal for BrdU-IP-chip datasets.

Although the data transformation proposed in [18] is not appropriate for BrdU-IP-chip data, we agree with their strategy of first transforming probe intensities onto an appropriate plane before further normalization. Thus, to remove intensity bias we have developed a data rotation method, robust to the nuances of both ChIP-chip and BrdU-IP-chip data, that we employ prior to the modified loess normalization step. We demonstrate our transformation on the "clean" *rpd*3Δ dataset, as it best displays the analytical issues associated with BrdU-IP-chip arrays; for analysis of the "noisier" *rpd*3Δ dataset see Additional file 2.

**Figure 3:**
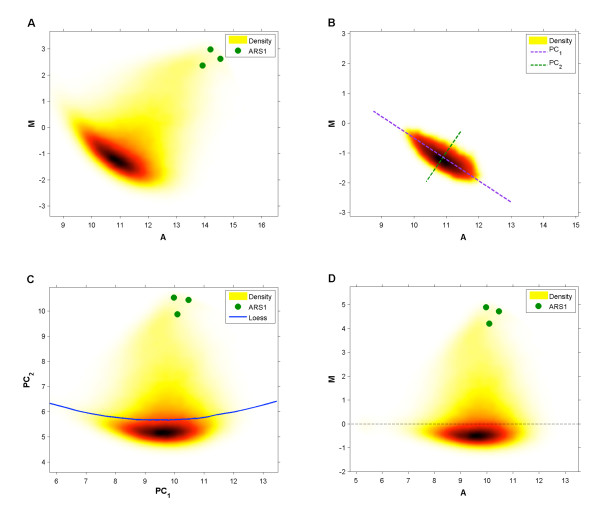
**Within-Array Normalization**. **(A) ***rpd*3Δ probes plotted in the MA plane (ARS1 probes are indicated with green dots). **(B) **The background probe subset plotted in the MA plane. The first and second principal component axes are used as the new set of axes in the data rotation. **(C) **Probes plotted in a modified MA plane after data rotation. A loess curve is then fitted to the probes within two standard deviations of the median M-value. **(D) **Probes plotted in the modified MA plane after loess normalization is complete.

An MA plot of the raw *rpd*3Δ data shows that the background probes (dark region), under the correct transformation, have a dense and relatively symmetric empirical M-distribution (Figure 3A). As shown in [18], this is a characteristic feature of ChIP-chip data, and thus the methods described below will also be applicable to such data. We propose a data transformation that takes advantage of, and searches for, a subset  of the *N *probes whose distribution best follows these characteristics. After the probes in  are identified we define a rotation line that follows their slope in the MA plane and adopt it as the x-axis for a modified MA plane.

To identify  we first search for the *D *densest subsets of probes  with sizes *k*_1 _= *N*/*D*, *k*_2 _= 2*N*/*D*,..., *k*_*D *_= *N*. Here, the density of a probe set is measured by the size of its minimum spanning tree in the MA plane; see methods for details. *D *is a parameter that determines the granularity of the algorithm (we use *D *= 100 here; for a more precise solution *D *can be increased at the expense of running time). Following this, we search for the smallest of the *D *subsets whose "symmetry" measure *R *(defined below) is greater than an experiment-specific cutoff *R*_*C *_(also defined below), and  is defined by this subset of probes.

To assess the symmetry of probes in the set  we calculate the first and second principal components,  and  respectively, of its probes in the MA plane, and define its symmetry measure *R*_*i *_by



where MST_*i *_denotes the minimum spanning tree of the subset, **1 **denotes the indicator of a set, and the cutoff *c*_*i *_is determined as the median of the -values of the set . We choose this subset size because we know *a priori *that less than 80% of probes are enriched in the experimental conditions being analyzed (this ensures that this subset contains primarily background probes; for other experimental conditions this subset size can be altered accordingly).

We define  as the set of size *k*_*j *_where



and *R*_*C *_= 2 × standard deviation of *R*_1_, *R*_2_,..., *R*_0.2*N*_. This choice is motivated by the observation that if *k*_*i *_is the size of the largest subset of size at most ||, then the values *R*_1_, *R*_2_, *R*,..., *R*_*i *_fluctuate at a value close to 0, whereas the values *R*_*i*+1_, *R*_*i*+2_,..., *R*_*D *_incrementally increase, as enriched probes are only included in the numerator of the ratio defining *R *(Additional file 3). The cutoff value *R*_*C *_is dependent on the *a priori *knowledge that at most 80% of all probes are enriched.

After  is identified, *all *probes are transformed into the plane whose *x *and *y *axes correspond to its first and second principle components, PC_1 _and PC_2 _respectively (Figure 3B). Following the rotation, the modified loess step proposed in [18] is applied to the data (with default parameter settings) and although the large numbers of enriched probes "pull" the loess curve away from the background distribution (Figure 3C), the data transformation ensures that the loess normalization does not distort the data and that the majority of the residual intensity bias is removed (Figure 3D).

**Figure 4:**
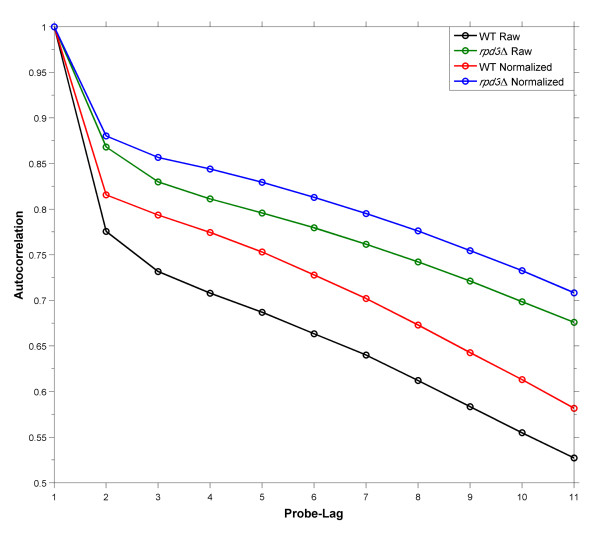
**Autocorrelation Analysis**. The correlation structure of the WT and *rpd*3Δ datasets before and after within-array normalization. y-axis: Spearman rank correlation. x-axis: lag, measured as number of probes along a chromosome.

The autocorrelation structure of probe M-values along the chromosome is inversely proportional to array noise and intensity bias and should increase when within-array normalization methods are carefully applied [18,20]. To assess our methods, we calculated the autocorrelations of both the WT and *rpd*3Δ datasets prior to and after application of our within-array normalization scheme at lags of 0 to 100 probes (corresponding to distances of 0 to ~300 base pairs). Figure 4 demonstrates that the proposed strategies reduce the intensity bias-related noise inherent in BrdU-IP-chip experiments. In addition the correlation structure of the WT data is worse than that of *rpd*3Δ. We think that this is due to the mutant array having a higher proportion of enriched probes, as noise appears to be more significant in non-enriched regions (compare Figures 2G & Additional file 4).

### Between-Array Normalization

#### Location Normalization

**Figure 5:**
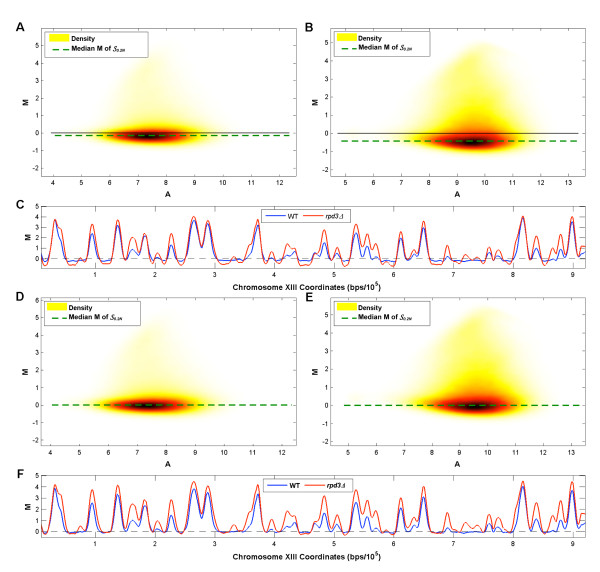
**Location Normalization**. **(A) **WT probes (after within-array normalization) plotted in the MA plane. The location parameter is the median M-value of . **(B) ***rpd*3Δ probes (after within-array normalization) plotted in the MA plane. **(C) **WT and *rpd*3Δ probes plotted in the chromosomal plane (chromosome XIII). **(D) **WT probes plotted in the MA plane after location normalization. **(E) ***rpd*3Δ probes plotted in the MA plane after location normalization. **(F) **WT and *rpd*3Δ probes plotted in the chromosomal plane (chromosome XIII) after location normalization.

When comparing the within-array normalized data across different experiments, further normalization is needed to correct for the fact that the M-values in  can have different locations. For example, when comparing the MA plots of WT and *rpd*3Δ after within-array normalization, the median is much lower in *rpd*3Δ (Figures 5A and 5B). When these data are plotted along the chromosome we see that the baseline of the *rpd*3Δ plot is artificially lower than that of WT (Figures 5C). If not corrected, this would result in errors when testing for differences between WT and *rpd*3Δ peaks. To correct for this, for each experiment we propose subtracting the median M-value of its  as calculated after within-array normalization (Figure 5D &5E). This strategy successfully normalizes the baseline across arrays, allowing comparisons between experimental conditions to be performed more accurately (Figure 5F).

#### Scale Normalization

We observe noticeable scale differences in the empirical M-distributions of experimental replicates. Before performing comparisons across various conditions, these experimental errors should be eliminated without removing differences attributable to true biological variation. We tested the existing strategies for scale normalization (MAD scaling and quantile normalization) and found that signal differences observed consistently between WT and *rpd*3Δ replicates, which we attribute to true replication landscape changes in *rpd*3Δ, are removed when either is applied (data not shown). With MAD scaling, differences between larger enrichment peaks are removed and with quantile normalization virtually all biological differences are eliminated.

**Figure 6:**
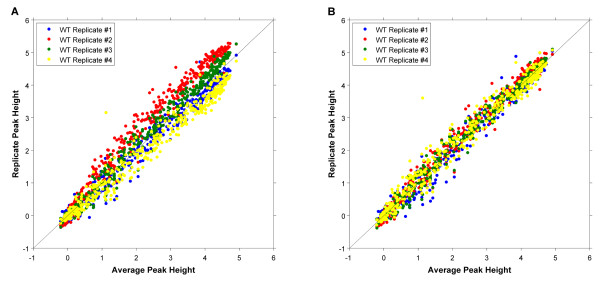
**Scale Normalization**. **(A) **Peak heights of each WT replicate, calculated before scale normalization, plotted against the average height across replicates. **(B) **Peak heights of each WT replicate, calculated after scale normalization, plotted against the average height across replicates.

Here we propose a modified quantile normalization procedure where the M-values of each set of replicates are normalized together [16], but not with replicates from other experimental conditions (e.g. the WT replicates are quantile normalized with one another separately from the *rpd*3Δ replicates). This forces replicates to better resemble each other (removing experimental error) without removing true biological differences. Figure 6A shows the peak heights from the four WT replicate datasets (for peak identification and quantification see below) plotted against their averages (before scale normalization). The scale differences result in discrepancies between replicate peaks with larger heights, which can be a source of false negatives when testing for peak height changes (e.g. the larger variation in peak heights results in a smaller t-statistic). Figure 6B shows that, when the modified quantile normalization strategy is applied, these size-dependent differences are removed.

### Peak Identification and Quantification

There are several ways in which peak identification and quantification can be performed. For example, we might average the observations from replicate experiments to get a single set of potential peaks for each experimental condition. Because there are often multiple peaks within a given enriched region that may be lost if averaging across replicates is used, we have found it better to identify peaks within each replicate, and then compare peaks across replicates (and perhaps conditions) using further alignment.

Several algorithms have been developed to identify enriched genomic regions in ChIP-chip data [13,18,20,22-27]. Many of these use Hidden Markov Models (HMMs) with two probe states, corresponding to enriched and non-enriched. Others have proposed simpler methods, such as setting an enrichment threshold based on the variability of the array noise [18]. Here we calculate a final enrichment cutoff, used below to identify positive signals, by taking advantage of the characteristics of the distribution of the M-values of background probes. We employ a strategy similar to that proposed in [24]: identify all probes whose M-values are less than the median of the set , as recomputed after within-array and between-array normalization, reflect them about this value, and set the cutoff to twice the sample standard deviation of the resulting distribution. We note that we could also use this distribution to provide p-values for ranking probes, but we do not explore this further here.

**Figure 7:**
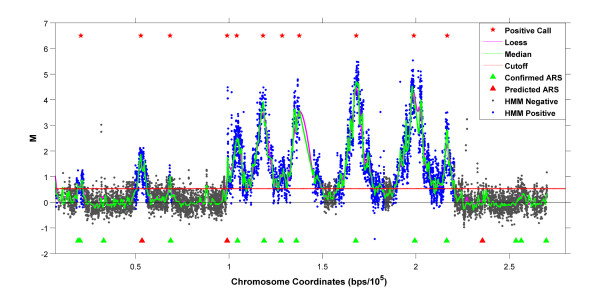
**Identification of Enriched Regions**. Peaks identified by the present method in a single replicate are marked with red stars. Probes in blocks called enriched by the HMM (posterior probability ≥ 0.5) are marked in blue and probes from non-enriched blocks are grey. Notice the agreement between the calls. Further details are provided in the text.

To identify individual replication peaks, we begin by fitting a loess curve to the normalized data on the chromosomal plane. Following this, a sliding window is applied to search for all regions with a continuous increase in smoothed M-values for at least 20 probes (~0.6 kbp) followed by a continual decrease for at least 20 probes (typical replication peaks are relatively symmetric about one apex; this choice can be changed for other types of data). We assign each peak a height equal to the median of the non-smoothed M-values within 500 bp of its apex and accept it as a potential positive if its height is greater than the enrichment cutoff (Figure 7).

After potential peaks have been identified for each experiment, we align them across replicates with a dynamic programming algorithm; see Methods for details. Following this, peaks present across all replicates are aligned with the known/predicted origins reported in the OriDB database [28]. This second alignment allows us to further confirm the validity of peaks with *a priori *knowledge of origin locations which, in turn, allows for an in-depth analysis of the chromosomal features surrounding the start point of each peak (see Methods for details).

### Validation

Typical BrdU experiments aim to identify genomic regions where there is evidence of replication activity, to determine its magnitude and to test if it is different in various cellular conditions. Below we validate our normalization and peak identification/quantification strategies both experimentally and statistically.

#### Peak Identification

We fitted an HMM [27] to the average normalized M-values of non-overlapping 1000 bp blocks of probes. The algorithm assigns to each such block the posterior probability of that block being in an enriched region. These probabilities can be used to rank and call potential enriched regions. Here, blocks with posterior probabilities ≥ 0.5 were called as enriched. A comparison of the HMM approach with the one presented here shows substantial agreement in positive peak calls (see Figure 7).

**Figure 8:**
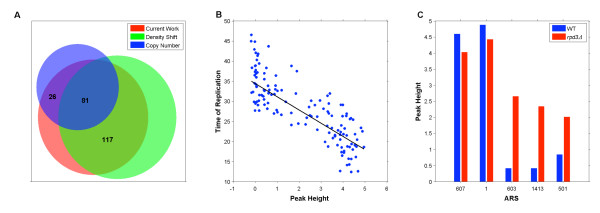
**Validation**. **(A) **251 origins are found to fire in this BrdU-IP-chip analysis as compared to the 290 identified in [30] and 141 in [29]. Of the 251 origins identified here 224 (89 percent) were identified in at least one of the other two studies. **(B) **142 WT peak heights (calculated here) plotted against their times of replication (as calculated in [31]). The Spearman Rank Correlation between peak heights and time of replication was found to be -0.78. **(C) **A comparison of WT and *rpd*3Δ peak heights shows significant increases (empirical Bayes t-test, *p *≤ 0.001) in *rpd*3Δ heights at origins ARS603, ARS1413 and ARS501 while the same analysis shows no change (empirical Bayes t-test, *p *> 0.001) at origins ARS607 and ARS1.

To validate experimentally our peak identification strategies, we compared the set of peaks identified here (in WT cells in HU) with those identified in two previous studies [29,30] where alternatives to the BrdU-IP-chip assay (density shift assay and copy number assay, respectively) were employed to map replication origins that fire in WT cells in HU. There were 141 origins found to fire in HU in [29] and 290 in [30]. Here we identified 251 origins as active in HU, with 107 (43 percent) overlapping with those identified in [29] and 198 (79 percent) with those identified in [30]. In total 224 (89 percent) of the origins we identified as active were found to fire in at least one of the two previous studies (Figure 8A).

#### Peak Quantification

To confirm that our array normalization and peak identification/quantification methods assign peak heights that are proportional to origin timing/efficiency, we compared the WT peak heights developed here to their times of replication (*T*_reps_) reported in [31]. We found that BrdU peak heights are significantly anticorrelated with *T*_reps_(Spearman's Rank Correlation of -0.78), indicating that high BrdU peaks are associated with early/efficiently firing origins, while lower BrdU peaks are associated with later firing less efficient origins (Figure 8B).

#### Strain Comparisons

To examine our ability to identify true biological variation across experimental conditions, we tested for peak height differences in the WT and *rpd*3Δ datasets (with empirical Bayes t-tests [32]) and compared these results to those in [19]. In this previous study three independent methods were used to compare the replication activity of five origins (ARS607, ARS1, ARS603, ARS1413 and ARS501) in WT and *rpd*3Δ cells. These three methods showed no significant difference between WT and *rpd*3Δ cells in origin firing times at ARS607 or ARS1 but found advanced origin firing in the *rpd*3Δ cells at ARS603, ARS1413 and ARS501. Comparisons of BrdU peak heights at these origins demonstrate significant peak height differences at ARS603, ARS1413 and ARS501 (*p *≤ 0.001 for all), but no significant differences at ARS607 or ARS1 (*p *= 0.122 and 0.21 respectively) (Figure 8C).

## Conclusion

The BrdU-IP-chip assay provides an effective technique to identify replication activity across the genome, and furthermore, the signal magnitude in these data is proportional to the percentage of cells in a culture that fire at each origin. As whole-genome analysis of replication dynamics continues to develop, a proper strategy for analyzing these and other datasets with similar characteristics is essential. Here we have shown that traditional strategies for dealing with expression and protein binding ChIP-chip experiments may be sub-optimal for the analysis of these types of data. We have developed strategies for both within-array and between-array normalization that are able to accommodate highly enriched datasets. Furthermore, we have presented peak identification, quantification and alignment tools that use *a priori *knowledge to remove both false positives and negatives. We have tested these methods both statistically and through a comparative analysis with previous studies to show that they are able to identify enriched regions correctly and that the array normalization and peak identification/quantification strategies are effective in detecting biologically meaningful changes in experiments performed under different conditions.

## Methods

### Modified k-MST Algorithm

Finding the *k*-vertex minimum spanning tree in a dataset of size *N *≥ *k *is an NP-hard problem known as *k-Minimum Spanning Tree *(*k*-*MST*). Instead of solving this directly, we employ a time-optimized version of an approximation algorithm aimed at identifying only the set of probes contained in the *k*-*MST *rather than the actual *k*-*MST *[33]. The algorithm proposed in [33] is polynomial in time, but current tiling array feature counts are now in the millions. To reduce its search space, and hence its running time, we have modified the algorithm in [33] by integrating an initial greedy step. First, probes are binned into cells of a uniformly spaced 128 × 128 grid (*I*) in the MA plane. Following this, cells of *I *(which we denote by *I*_*ij*_, 1 ≤ *i*, *j *≤ 128) and their probes are added to a set *C *in descending order of the number of probes (|*I*_*i, j*_|) they contain, until *k *- *N*/*D *≤ |*C*| ≤ *k*, where |*C*| is the total number of probes in the cells of *C*.

Following this, "layers" of cells neighboring *C *are added to a set *Q *until |*C*| + |*Q*| ≥ *k*. More precisely, when a new neighboring "layer" is to be added to *Q*, its cell set is defined as



We then alter the algorithm in [33] so that all probes in *C *are included in the final *k*-probe solution and the search space for the additional *k *- |*C*| probes is constrained to the cells in *Q*. In [33] the authors employ a set of grids *G*_0_, *G*_1_,..., *G*_*n *_whose cells each have corresponding list *L*. To ensure the above constraints are followed, we initialize the lists corresponding to the cells of the finest grid, *G*_0 _(a 256 × 256 grid here) as follows:



where *x*_0 _and *m *are the width of, and number of probes in, the cell corresponding to *L*, respectively. After *L *has been computed for each of the cells in *G*_0_, the algorithm proceeds as described in [33], with the following modifications: (i) for a larger cell *c *and corresponding list *L*, if *r *of the probes in *c *are contained in *C*, *L*(*p*) = ∞ for *p *<*r*; (ii) *L*(*r*) is calculated by merging all lists corresponding to subcells of *c *that are contained in *C*, and (iii) for *r *<*q *≤ *k*, *L*(*q*) is calculated by merging *L*(*r*) with all lists corresponding to subcells of *c *that are not contained in *C*. After completion, the final set of *k *probes used for subsequent analysis is that corresponding to *L*(*k*) for the 1 × 1 grid *G*_*n *_(see [33] for further details).

### Peak Alignment Across Experiments

To identify peaks that are present across a set of *r *replicates we perform a multiple global alignment on their replicate-specific locations using a version of the Needleman-Wunsch algorithm [34] similar to the one described in [35]. Each element *A *of the alignment set  is represented in the form of a sequence of tuples:



The first element *C *of each tuple defines the chromosomal origin of a peak. The second element in the tuple, {(*E*_1_, *L*_1_), (*E*_2_, *L*_2_),..., (*E*_*v*_, *L*_*v*_)} say, is a set of tuples consisting of experiment labels (*E*) and corresponding chromosomal locations (*L*) of peaks that are identified as aligned in experiments *E*_1_,..., *E*_*v*_. The method starts with the peak locations identified above in each experiment; the peaks in the *j*th experiment can be represented in the form



The algorithm proceeds by successively calculating all pairwise alignments and alignment distances between sequences in  with the Needleman-Wunsch algorithm, each time replacing the most similar pair with its alignment:



where |Alignment(.,.)| is equal to the bottom right hand corner of the Needleman-Wunsch distance matrix calculated during an alignment. During an alignment, if peaks (*C*, {(*E*, *L*)}) and (*C'*, {(*E'*, *L'*)}) from two inputs are deemed close enough, they are merged into a single peak (*C"*, {(*E"*, *L"*)} in the output alignment. This new peak has chromosomal origin *C" *= *C' *= *C*, and {(*E"*, *L"*)} = {(*E*, *L*)} ∪ {(*E'*, *L'*)}. Peaks that are not deemed close enough are not merged and their values are inserted separately into the new alignment.

It remains to define the distance measure to be used in the Needleman-Wunsch algorithm. For peaks *P *= (*C*, {(*E*_*u*_, *L*_*u*_)}) and *P' *= (*C'*, {(, )}), we set



The gap penalty is the maximum distance permitted between two aligned peaks. Here we set it to 2000, as an empirical analysis across experiments showed that several large corresponding peaks had coordinate differences up to 1700 bp.

### Peak Alignments With Known/Predicted Origins

We align peaks with known/predicted origin locations (as listed in OriDB) to remove some false positives and to determine the precise genomic loci that each BrdU peak emanates from. OriDB lists origins in one of three categories: confirmed (confirmed with an ARS stability assay), likely (inferred in two or more experiments) or dubious (inferred in only one experiment). Based on the assumption that peaks are more likely associated with confirmed than dubious origins, we perform peak/origin alignments in a three-step process designed to align peaks with the highest ranking origin in their vicinity.

### Alignment

We begin with the final sequence of peak locations (*A *= ) and three sets of chromosomally ordered origin locations *O*_*C*_, *O*_*L *_and *O*_*D *_(corresponding to confirmed, likely and dubious origin sets, respectively). An origin location in one of these sets is a triplet *O *= (*O*^*ch*^, *O*^*s*^, *O*^*e*^) giving its chromosome, its starting coordinate and its ending coordinate, respectively. The alignment proceeds as follows:



and the final set of peak/origin pairs are held in the set *T*.

### Distance Function

Although we employ the same gap penalty as during the alignment of replicates described above, we alter the distance function to reflect the fact that peaks located between the start and end coordinates of an origin should have a distance of zero from that origin. Thus, we define the distance between a peak *P *= (*C*, {(*E*_*u*_, *L*_*u*_)}) and an origin *O *as follows:



## Authors' contributions

SRVK developed the computational methods with assistance from ST, CJV performed the biological experiments and OMA provided biological insights. SRVK and ST wrote the paper. All authors read and approved the final manuscript.

## Supplementary Material

Additional file 1 **Testing ChIP-chip Normalization Methods on Noisy Data**. Illustration of method proposed in [18] for normalization of "noisy" BrdU-IP-chip data. **(A) ***rpd*3Δ probes (from the "noisy" *rpd*3Δ dataset) plotted in the MA plane (ARS1 probes are indicated with green dots). **(B) **Each probe is plotted in the MA plane and a line of best fit, which should run parallel to the slope of the background distribution, is employed as the x-axis on the modified MA plane. **(C) **Probes transformed onto the modified MA plane. Following this transformation a loess line is fitted to probes within two standard deviations of the median M-value. **(D) **Probes plotted in the modified MA plane after the final loess normalization step. Click here for file

Additional file 2 **Within-Array Normalization on a "Noisy" *rpd3Δ *Dataset**. **(A) **Probes from the "noisy" *rpd*3Δ dataset plotted in the MA plane. **(B) **The background probe subset plotted in the MA plane. The first and second principal component axes are used as the new set of axes in the data rotation. **(C) **Probes plotted in the modified MA plane after data rotation. After this rotation a loess curve is fitted to the probes within two standard deviations of the median M-value. **(D) **Probes plotted in the modified MA plane after the modified loess normalization. Click here for file

Additional file 3 **Symmetry Measurements**. During within-array normalization non-enriched probes are identified as the largest set with a symmetry measure *R *≤ *R*_*C *_= 2 × standard deviation of *R*_1_, *R*_2_,..., *R*_0.2*N*_. *R *fluctuates about 0 while only background probes are included in its calculation. When enriched probes begin to be included in its calculation, *R *incrementally increases. Click here for file

Additional file 4 ***rpd*3Δ probes plotted in the chromosomal plane**. Raw M-values of *rpd*3Δ probes plotted in the chromosomal plane (chromosome XIII shown here). Click here for file
