# Supplementary figures and images for: Strategies for analyzing highly enriched IP-chip datasets
Source: BMC Bioinformatics. 2009 Sep 22;10:305. doi: 10.1186/1471-2105-10-305 (PMC2759964; doi:10.1186/1471-2105-10-305)

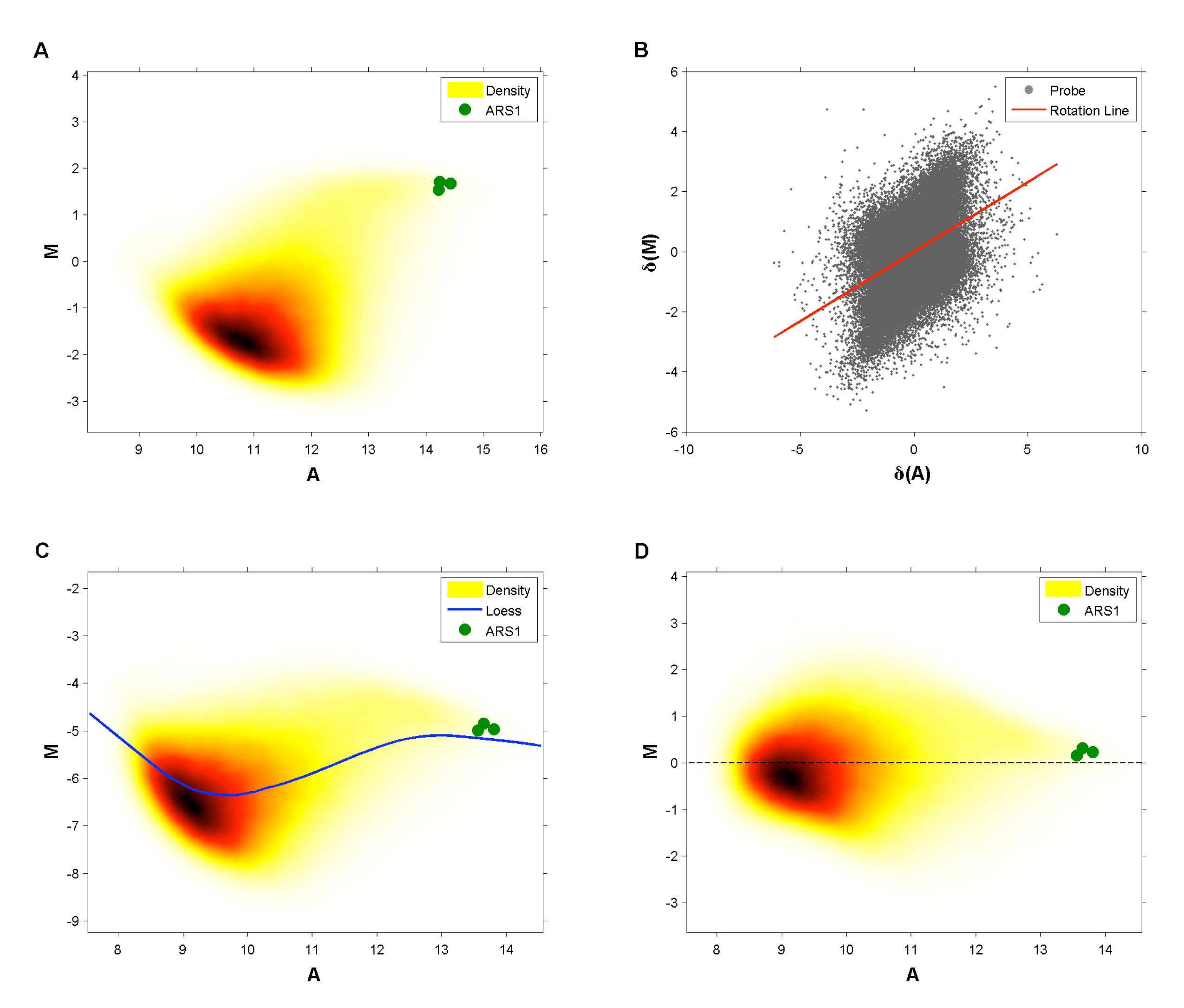

Supplement: Additional file 1 — Testing ChIP-chip Normalization Methods on Noisy Data. Illustration of method proposed in [18] for normalization of "noisy" BrdU-IP-chip data. (A) rpd3Δ probes (from the "noisy" rpd3Δ dataset) plotted in the MA plane (ARS1 probes are indicated with green dots). (B) Each probe is plotted in the MA plane and a line of best fit, which should run parallel to the slope of the background distribution, is employed as the x-axis on the modified MA plane. (C) Probes transformed onto the modified MA plane. Following this transformation a loess line is fitted to probes within two standard deviations of the median M-value. (D) Probes plotted in the modified MA plane after the final loess normalization step. [file 1471-2105-10-305-S1.tiff]

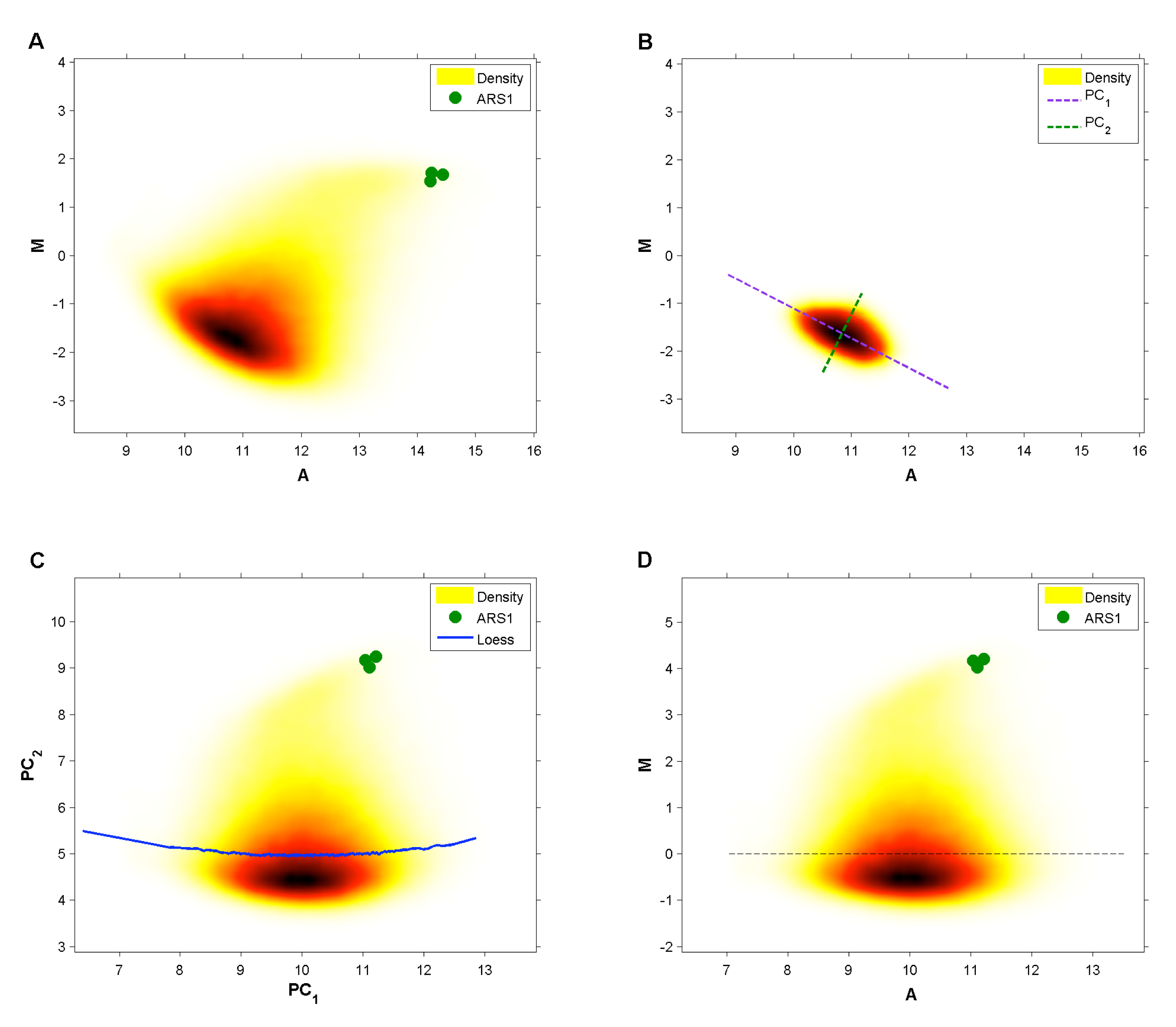

Supplement: Additional file 2 — Within-Array Normalization on a "Noisy" rpd3Δ Dataset. (A) Probes from the "noisy" rpd3Δ dataset plotted in the MA plane. (B) The background probe subset plotted in the MA plane. The first and second principal component axes are used as the new set of axes in the data rotation. (C) Probes plotted in the modified MA plane after data rotation. After this rotation a loess curve is fitted to the probes within two standard deviations of the median M-value. (D) Probes plotted in the modified MA plane after the modified loess normalization. [file 1471-2105-10-305-S2.tiff]

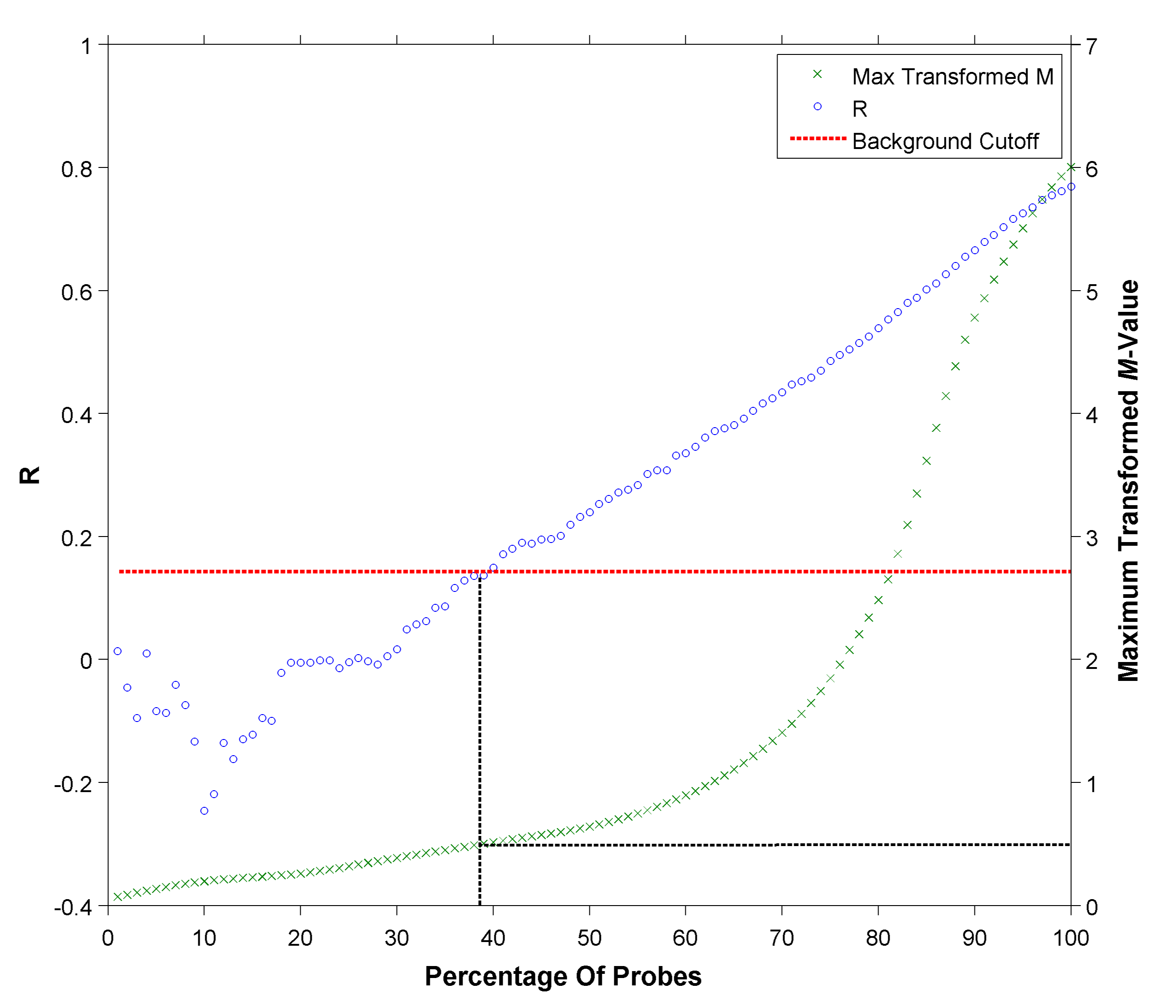

Supplement: Additional file 3 — Symmetry Measurements. During within-array normalization non-enriched probes are identified as the largest set with a symmetry measure R ≤ RC = 2 × standard deviation of R1, R2,..., R0.2N. R fluctuates about 0 while only background probes are included in its calculation. When enriched probes begin to be included in its calculation, R incrementally increases. [file 1471-2105-10-305-S3.tiff]

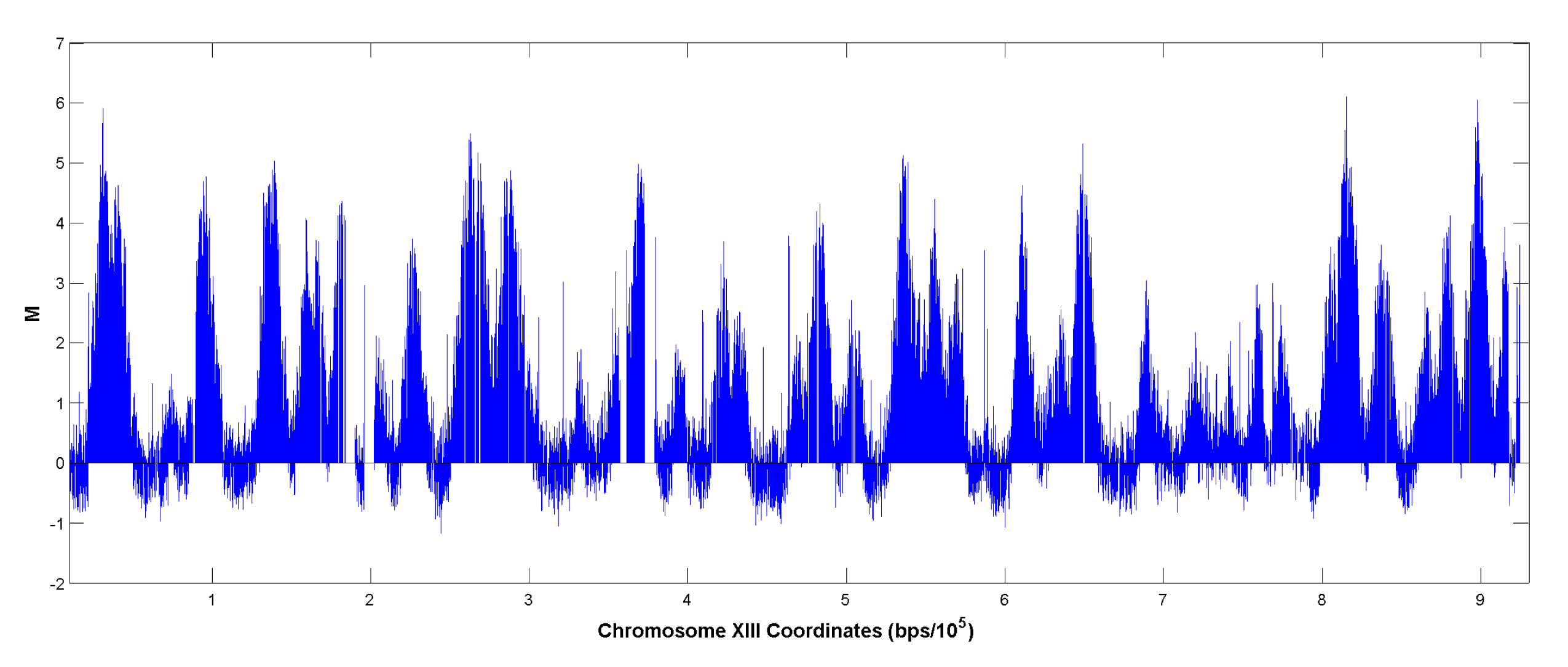

Supplement: Additional file 4 — rpd3Δ probes plotted in the chromosomal plane. Raw M-values of rpd3Δ probes plotted in the chromosomal plane (chromosome XIII shown here). [file 1471-2105-10-305-S4.tiff]
